# Reducing severe fatigue in patients with diffuse glioma: a study protocol for an RCT on the effect of blended cognitive behavioural therapy

**DOI:** 10.1186/s13063-022-06485-5

**Published:** 2022-07-15

**Authors:** Jantine Geertruida Röttgering, Linda Douw, Philip C. de Witt Hamer, Mathilde C. M. Kouwenhoven, Tom Würdinger, Peter M. van de Ven, Louise Sharpe, Hans Knoop, Martin Klein

**Affiliations:** 1 Amsterdam UMC location Vrije Universiteit Amsterdam, Medical Psychology, De Boelelaan 1117, Amsterdam, The Netherlands; 2 Cancer Center Amsterdam, Brain Tumor Center, Amsterdam, The Netherlands; 3 Amsterdam UMC location Vrije Universiteit Amsterdam, Anatomy and Neurosciences, De Boelelaan 1117, Amsterdam, The Netherlands; 4 Department of Radiology, Massachusetts General Hospital, Athinoula A. Martinos Center for Biomedical Imaging, 149 13th Street, Boston, MA 02129 USA; 5 Amsterdam UMC location Vrije Universiteit Amsterdam, Neurosurgery, De Boelelaan 1117, Amsterdam, The Netherlands; 6 Amsterdam UMC location Vrije Universiteit Amsterdam, Neurology, De Boelelaan 1117, Amsterdam, The Netherlands; 7 Amsterdam UMC location Vrije Universiteit Amsterdam, Epidemiology and Data Science, Amsterdam, The Netherlands; 8 The School of Psychology, University of Sydney, Sydney, NSW Australia; 9 Amsterdam UMC location University of Amsterdam, Medical Psychology, Meibergdreef 9, Amsterdam, The Netherlands; 10 Amsterdam Public Health Research Institute, Expert Center for Chronic Fatigue, Amsterdam, The Netherlands

**Keywords:** Psychosocial intervention, Glioma, Fatigue, Blended cognitive behavioural therapy, Web-based, Digital health, Online intervention, Cancer, Health-related quality of life

## Abstract

**Background:**

Fatigue is the most frequent and burdensome symptom of patients with diffuse glioma. It is closely linked to decreased health-related quality of life and symptoms such as depression and sleep disturbances. Currently, there is no evidence-based treatment that targets severe fatigue in patients with brain tumours. Cognitive behavioural therapy is aimed at fatigue-maintaining beliefs and behaviour. This therapy has been proven effective in reducing severe fatigue in cancer survivors and patients with multiple sclerosis. A blended therapy program combines sessions with a therapist with therapist-guided web-based therapy modules. The aim of this randomized controlled trial is to determine the efficacy of blended cognitive behavioural therapy in treating severe fatigue in patients with diffuse glioma.

**Methods:**

We will include a maximum of 100 patients with diffuse glioma with clinically and radiologically stable disease and severe fatigue (i.e. Checklist Individual Strength, subscale fatigue severity ≥ 35). Patients will be randomized to blended cognitive behavioural therapy or a waiting list condition. The 12-week intervention *GRIP on fatigue* consists of five patient-therapist sessions and five to eight individualized web-based therapy modules supported by email contact. The primary outcome measure is fatigue severity. Secondary outcome measures include sleep quality, health-related quality of life, depression, anxiety, functional impairment and subjective and objective cognitive functioning. Primary and secondary outcome measures will be assessed at baseline and after 14 and 24 weeks. Magnetoencephalography and MRI will be used to evaluate potential biomarkers for intervention success. This trial has a Bayesian design: we will conduct multiple interim analyses to test for efficacy or futility of the trial. This is the first trial within the *GRIP trial platform*: a platform developing four to five different interventions for the most common symptoms in patients with diffuse glioma.

**Discussion:**

The results of the *GRIP on fatigue* trial will provide information about the efficacy of this intervention on fatigue in patients with diffuse glioma. Multiple other outcomes and possible predictors of treatment success will also be explored.

**Trial registration:**

Netherlands Trial Register NL8711. Registered on 14 June 2020.

**Supplementary Information:**

The online version contains supplementary material available at 10.1186/s13063-022-06485-5.

## Background

Patients with diffuse glioma are a distinct group within the cancer population, characterized by continuous tumour growth leading to inevitable death. Malignant gliomas are the most common primary brain tumour with an incidence of 6 per 100,000 in Europe [1]. During the disease trajectory, patients experience a multitude of symptoms such as fatigue, cognitive impairment and neurological deficits, resulting in a high symptom burden and impaired health-related quality of life (HRQOL) [2–4].

Fatigue is one of the most frequently reported symptoms in patients with a brain tumour. Cancer-related fatigue (CRF) is “a distressing, persistent, subjective sense of physical, emotional, and/or cognitive tiredness or exhaustion related to cancer that is not proportional to recent activity and interferes with usual functioning” [5]. The prevalence of fatigue in diffuse glioma patients varies between studies; it is reported to affect up to 96% of patients in different stages of the disease [6–10]. In a study of long-term survivors with low-grade glioma, 40% of the survivors reported to be severely fatigued [11]. Additionally in a qualitative study, patients with diffuse glioma described their tiredness as the most severe of the multiple symptoms that they experience [12].

The aetiology of fatigue in patients with a brain tumour is complex and poorly understood. Demographic, biomedical, neuropsychological, psychosocial and behavioural factors may contribute to the origin and persistence of fatigue [13]**.** Fatigue is associated with cognitive complaints and various other symptoms in different symptom clusters, such as depression, anxiety and sleep–wake disturbances, and has a negative influence on role functioning [10]. These different clusters of symptoms have a large impact on everyday life of patients and is linked to a decreased HRQOL [7, 10, 13, 14].

No evidence-based intervention is currently available for the treatment of fatigue in patients with a brain tumour, even though it is thought that effective treatment could improve HRQOL [3, 13]. A 2016 Cochrane review on treating fatigue in primary brain tumour patients could only identify one randomized controlled trial (RCT) that restricted inclusion to severely fatigued patients and had fatigue as the primary outcome measure [8]. This trial was conducted by our group and did not show an effect of the psychostimulant modafinil on fatigue [15]. Inclusion and follow-up rates were lower than expected, because patients were reluctant to take more medication or they experienced side effects. However, several trials that did not limit inclusion to severely fatigued brain tumour patients, but also included brain tumour patients without severe fatigue, have determined positive effects of cognitive rehabilitation and pharmacological treatment on fatigue [16–20]. Despite the lack of evidence-based interventions, the majority of patients seek treatment for their symptoms through complementary medicine [21], indicating a unmet need to alleviate symptoms.

The treatment of CRF has been studied more extensively in non-central nervous system cancer survivors and in palliative cancer patients. Systematic reviews have indicated that Cognitive behavioural therapy (CBT) can reduce fatigue in these patient groups [20, 22]. Fatigue-specific CBT is based on the assumption that cancer treatment and cancer itself can trigger fatigue, but that factors such as sleep disturbances and unhelpful thoughts can contribute to the persistence of fatigue [23, 24]. Treatments targeting such factors may therefore be promising interventions to alleviate fatigue in brain tumour patients. A recent RCT conducted by our group in severely fatigued cancer patients, who were treated with palliative intent, showed a significant reduction of fatigue after a 12-week face-to-face CBT intervention [25]. Cancer survivors who receive CBT for cancer-related fatigue not only report a reduction in fatigue, but also report less cognitive disability and concentration problems [26]. Also, the blended version of the CBT intervention showed a significant reduction of fatigue in breast cancer survivors [27]. CBT as a treatment for fatigue also seems promising in patients with neurological disease. It has shown to be effective in reducing severe fatigue in patients with multiple sclerosis [28] and is studied in treating post-stroke fatigue [29, 30]. Whether fatigued patients with diffuse glioma could benefit from CBT to resolve or reduce fatigue remains to be determined.

The primary objective of this RCT is to determine whether a 12-week therapist-guided blended CBT (BCBT) intervention will reduce severe fatigue post-intervention in patients with diffuse glioma compared to a waiting list condition (WLC). Secondarily, we will determine whether the intervention results in an improvement of sleep quality, HRQOL, depression, anxiety, functional impairment and subjective and objective cognitive functioning. In addition, we will evaluate invested time by patients and therapists and patients’ satisfaction with the treatment and the online platform. Furthermore, we will investigate possible biomarkers for treatment response with exploratory measurements (e.g. advanced neuroimaging and neurophysiology [31–35]).

## Methods

This intervention, the *GRIP on fatigue trial*, is part of the *Guarding Quality of Survivorship (GRIP) trial platform.* The aim of this platform is to develop four to five different interventions for the most common symptoms in patients with diffuse glioma, such as fatigue, cognitive deficits, anxiety and reduced physical fitness [3]. This trial is the first to be launched within this platform. The other trials are under development.

### Recruitment, screening and randomization

Patients visiting the CCA Brain Tumour Center Amsterdam (Amsterdam University Medical Centers)—a tertiary referral centre for patients with brain tumours—will be screened by their treating health care professional (e.g. neurologist, neurosurgeon, psychologist). Patients diagnosed with histologically confirmed diffuse glioma (WHO grade 2, 3 or 4) with clinically and radiologically stable disease and an expected survival of at least three months are eligible for inclusion. A set of laboratory tests is performed: haemoglobin, CRP, sedimentation rate, leukocyte count and differentiation, thrombocyte count, TSH, LDH, ASAT, ALAT, gamma-GT, sodium, potassium, urea, creatinine and glucose. These laboratory results will be screened to exclude medically treatable causes of fatigue.

Patients complete screening questionnaires to check for eligibility: Checklist Individual Strength (CIS) [36] and the Beck Depression Inventory Primary Care (BDI-PC) [37]. See Table 1 for all inclusion and exclusion criteria. The presence of severe fatigue is a criterion for inclusion and is reflected by a score ≥ 35 on the CIS subscale fatigue severity (CIS-fatigue) [36]. The Mini-International Neuropsychiatric Interview-depressive disorder [38] will be conducted over the phone if the patient has a BDI-PC score ≥4. If the criteria of a depression are met, the patient will not be included and will be referred for diagnostics and treatment. Demographic characteristics and disease-related determinants will be collected from the medical file. Comorbidities are assessed by the researcher using the Cumulative Illness Rating Scale [40].

**Table 1:** Inclusion and exclusion criteria

Inclusion criteria	Exclusion criteria
(1) Histologically confirmed diffuse glioma WHO grade 2, 3 or 4	(1) Treatable somatic cause that could explain the presence of severe fatigue (other than the underlying disease and its treatment)
(2) Age ≥ 18 years	(2) Primary sleep disorders previously diagnosed by a physician
(3) CIS subscale fatigue severity ≥ 35 (36)	(3) Current treatment by a psychiatrist or psychologist for a psychiatric disorder
(4) Expected survival of at least three months, as determined by treating clinician	(4) Suspected depression by screening with BDI-PC≥4 [37] and The Mini-International Neuropsychiatric Interview depressive disorder [38]
(5) No oncological treatment for at least two months prior to inclusion	(5) Pregnancy or given birth in the past three months
(6) No signs of radiological or clinical tumour progression at the time of inclusion	(6) Pharmacological treatment for fatigue, started in the past three months
(7) Able to speak, read and write Dutch	(7) Karnofsky Performance Status score <70 [39]
(8) Access and ability to use the internet	(8) Corticosteroid use
(9) Written informed consent	

Abbreviations: *CIS* Checklist Individual Strength, *BDI-PC* Beck Depression Inventory – Primary Care

After signing for informed consent and completing the screening procedure, the patient will be randomized to the BCBT intervention or to the WLC group with the use of the web-based computer program, Castor EDC [41]. This program automatically randomizes the patient to one of the two conditions. To keep the number of patients randomized to the two arms balanced throughout the trial, the program uses block randomization with a block size of two and four in random order. A block of two consists of one randomization to the BCGT group and one randomization to the WLC group in random order. A block of four consists of two BCGT randomisations and two WLC group randomisations in random order [42]. After the screening process, the patient is informed on the outcome of the randomization.

### Assessments and procedures

**Fig. 1:**
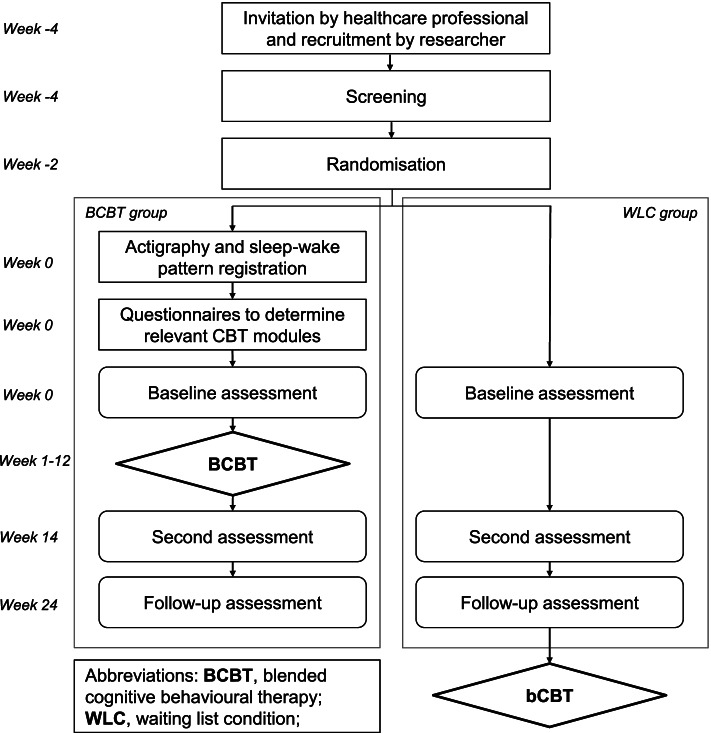
Flowchart of the trial

There are three trial assessments (see Fig. 1 for the flowchart): a baseline assessment after randomization and before the start of the intervention, a second assessment 14 weeks after baseline, and a follow-up assessment 24 weeks after baseline. The baseline and second assessments consist of web-based questionnaires, which are completed at home, and several measurements in the hospital (e.g. MRI, magnetoencephalography, neurocognitive testing and neurological examination). The questionnaires and measurements are explained in further detail at the endpoints section. The follow-up assessment only consists of web-based questionnaires.

All patients will be treated in concordance with national and regional glioma clinical practice guidelines of the National Dutch Working Group on Neuro-Oncology [1]. Patients are asked not to follow any other interventions or use pharmaceuticals aimed at treating fatigue during trial participation. Patients assigned to the WLC group are on a waiting list and will have the opportunity to do the BCBT program after the follow-up period (see Fig. 1). Patients can withdraw from the study at any time for any reason. The researcher can withdraw patients from the study in case of tumour progression or incorrect enrolment. Patients that drop out or are withdrawn are asked to still conduct the second and follow-up assessments.

### Intervention: GRIP on fatigue

*GRIP on fatigue* is a multimodal Dutch intervention with therapist sessions and online modules with therapist feedback. BCBT for CRF in patients with a brain tumour is directed at the beliefs and behaviour of the patient that contribute to the persistence of fatigue.

The intervention lasts 12 weeks. There are five patient–therapist sessions, of which those at the start and finish of the intervention are face to face. The other sessions can be either face-to-face or via secure video consultation, depending on the patients’ preferences. This is combined with five to eight web-based therapy modules delivered via the web portal Minddistrict (www.minddistrict.nl, CE marking 2017/590-01) hosted by Intermax (SO27001 and NEN-7510 certified). The patient is supported by messaging and feedback from the therapist within the online portal. This portal is available via a web browser on the computer or via an application for tablet and mobile phone.

The information and assignments are based on several evidence-based CBT interventions in different populations targeting fatigue by our research group [27, 43, 44]. These interventions have been adapted by experts on brain tumours and CRF to target patients with a brain tumour. The intervention is based on the assumption that having a brain tumour and/or the operation and treatment may initially trigger fatigue, but that other factors such as sleep disturbance, anxiety, physical inactivity, and dysfunctional thoughts about fatigue are responsible for the persistence of fatigue [13, 23, 43]. The module on fear of recurrence is based on the Conquer Fear program, which has shown to be effective in reducing fear of recurrence in comparison to relaxation training [45]. It is expected that patients spend approximately one hour per day on completing the online intervention.

As there are many individual differences in fatigue maintaining cognitive-behavioural factors, patients who are treated with BCBT undergo assessments to determine the relevant fatigue-maintaining factors and select the right treatment modules addressing these factors. Those instruments form an integral part of the intervention and include a set of questionnaires, actigraphy for fourteen consecutive days and simultaneously keeping a sleep–wake diary on seven consecutive days before the intervention starts. With actigraphy, two different activity patterns will be distinguished: relatively active or low active. The instruments are used for tailoring the treatment, for example the module *Activity regulation* will be personalized with regards to the activity pattern of the patient. Table 2 gives an overview of all modules of the intervention and their content. Some of the modules are optional and only indicated with specific scores on the relevant questionnaires.

**Table 2:** Content of the online modules and assessments

Online modules	Assessments
*1. Introduction and goals* The patient formulates positive and tangible goals, which consist of activities they want to do when no longer severely fatigued.	
*2. Sleep and rest* The patient makes a sleep schedule and keeps an online diary with sleep and wake times. A regular sleep–wake cycle and sleep hygiene are discussed. Instructions are given on how to improve these.	- Sickness Impact Profile (subscale sleep and rest) [46] - Registration of bedtime, wake-up time and sleep during the day for seven consecutive days
3. *Fatigue-related cognitions* Loss of control over fatigue symptoms and thoughts, fatigue catastrophizing and dysfunctional thoughts are assessed. The patient does exercises to address and change their dysfunctional thoughts and keeps an online diary about these thoughts. Patients learn to focus less on fatigue.	- Fatigue Catastrophizing Scale [47] - Illness Management Questionnaire factor III [48] - Self Efficacy Scale Fatigue [49]
*4. Activity regulation* The patient with a ‘relatively active’ activity pattern learns to distribute activities more evenly. Then both ‘relatively active’ and ‘low active’ patients systematically increase their physical activity with a graded activity program with walking or cycling. They track their daily progress in an online diary. They learn how to solve problems with activity regulation. The module aims to change activity-impeding beliefs and increase the physical activity level of patients.	- With actigraphy (actometer around the ankle for 14 consecutive days) the level of activity is objectified [50]. The activity pattern will be rated as ‘Low active’ or ‘Relatively active’.
*Submodule 4A: Regulation of social activities (optional)* The relationship between cancer, fatigue and a reduction of social activities as well as cognitions about social activities are assessed. The patient increases his/her social activity level.	- Sickness Impact Profile (*indication for this optional submodule: score subscale social activities ≥ 100*) [46]
*Submodule 4B: Regulation of mental activities (optional)* The patient learns about cognitive deficits and how to deal with them. The patient increases their mental activity level.	- Checklist Individual Strength *(indication for this optional submodule: score subscale concentration ≥ 18)* [36]
*Submodule 4C: Going back to work (optional)* The patient makes a plan to return to work or increase working hours.	*(indication for this optional submodule: if the patient has set a goal to return to work or increase working hours)*
*5. Fear of disease progression (optional)* Thoughts and situations that trigger fear regarding the future or tumour growth are assessed. The patient learns to be more accepting towards anxious feelings and to handle these feelings with exercises based on detached mindfulness, meta-cognitive therapy and exposure.	- Fear of Progression Questionnaire *(indication for the module: score ≥ 34)* [51] - Beck Anxiety Inventory *(indication for the module: score ≥ 36*) [52]
*6. Social support (optional)* Reactions of the partner and significant others to fatigue are assessed. Perceived discrepancy between actual and desired social support, experiences with negative social interactions and unrealistic expectations of others are assessed. The goal of this module is to support emotional independence of others and to become more assertive, as far as fatigue is concerned.	- Social Support List, subscale discrepancy *(indication for the module: score ≥ 50*) and subscale negative interactions (*indication for the module: score ≥ 14*) [53]
*7. Living with a brain tumour (optional)* This module focuses on uncertainty about the future and how one can deal with the fact that one has an incurable disease. Several elements from meaning-centred psychotherapy, well-being therapy and writing therapy are used to help the patients to deal with the disease trajectory.	- Illness Cognition Questionnaire, subscale acceptance (*indication for the module: score* ≤ 12) and subscale helplessness (*indication for the module: score ≥ 14*) [54] - Impact Event Scale, subscale avoidance (*indication for the module: score ≥ 10*) and subscale intrusion (*indication for the module: score ≥ 10*) [55]
*8. Realizing goals* The patient looks back at the goals set in the first module and makes a plan to realize these goals. The intervention is evaluated.	

The intervention will be provided by two to four trained and experienced cognitive-behavioural therapists, working at the Expert Center for Chronic Fatigue of the Amsterdam UMC, a tertiary treatment centre for fatigue. Every patient is assigned to one therapist, based on the availability of the therapist. The therapists are trained in delivering interventions on fatigue and this intervention specifically. They will be supervised every two weeks by a therapist with experience in BCBT in patients with a brain tumour.

### Primary and secondary endpoints

The primary outcome measure is the CIS-fatigue score at the second assessment, 14 weeks after baseline. The eight CIS-fatigue items are rated on a seven-point Likert scale with a total score ranging from 8 to 56. A score ≥ 35 indicates the presence of severe fatigue [36]. See Additional file 1 for an overview of all primary, secondary and exploratory assessments.

The set of secondary outcomes consists of several symptoms, quality of life and level of functioning measured by questionnaires at baseline, after 14 weeks and in follow-up, including depression, HRQOL and anxiety.

Neurocognitive functioning is tested using a standardized clinical test battery that is normally used for clinical care to evaluate preoperative and postoperative cognitive status. It consists of widely used standardized psychometric instruments with published normative data, such as the Stroop Color Word Test [56] and the Rey Complex Figure Test [57].

Furthermore, data about the usage of the online platform are collected, such as the number of logins, start and finish date of a module, completion date of the diaries and the amount of time spent on the platform. Therapists will register the time they invest per patient. Patients’ expectations of intervention outcome [58] and their satisfaction with the intervention and the platform [59] will be assessed before and after the intervention, respectively.

### Exploratory measurements

At baseline and after 14 weeks several exploratory measurements will be conducted, see Additional file 1 for an overview. These measurements are used to identify possible biomarkers for intervention success.

Brain connectivity and network topology will be assessed with magnetoencephalography, resting-state functional MRI, and diffusion MRI. Magnetoencephalography provides a non-invasive tool to study the brain’s neuronal networks and functional connectivity [60, 61]. Properties of baseline network topology of the brain may predict outcome of CBT [34, 35].

Furthermore, patients will undergo neurological examination, using the Neurologic Assessment in Neuro-Oncology Scale [62]. This is a standardized method of neurological physical examination in neuro-oncology patients and an objective clinician-reported outcome of neurologic function.

### Trial and data management

Personal data will be handled confidentially and data collection will comply with the EU General Data Protection Regulation and the Dutch Act on Implementation of the General Data Protection Regulation. Participants will be assigned a study identification number, which will be used on all research documents. This number is stored in a master file, only accessible to the principal investigator, co-investigators and study monitor. The web-based application Castor EDC will be used for coded data entry and storage [41]. All the questionnaires are conducted digitally with the use of this platform and automatically stored. Data concerning the usage of the online platform Minddistrict are stored within the online platform itself. Neuropsychological assessments and informed consent forms will be stored in locked file cabinets at the Department of Medical Psychology of the Amsterdam UMC. All magnetoencephalography and MRI files will be stored on a network server at the hospital. Data will be stored until 15 years after the completion of the project. This study will be subject to independent on-site monitoring in accordance with the Dutch Federation of University Medical Centers quality assurance advice regarding research involving human subjects.

### Sample size calculation and Bayesian design

A multicentre trial by our group on a 12-week BCBT intervention for fatigue among severely fatigued patients with advanced cancer showed that 14 weeks after baseline, patients in the intervention group reported significantly lower CIS-fatigue scores compared to patients receiving care as usual (−7.22, 97.5% CI −12.73 to −1.72; *p*=0.003, Cohen’s *d*=0.72) [25]. Based on these outcomes we anticipated a standardized effect size (Cohen’s *d*) of 0.7 on the CIS-fatigue.

The required number of patients was calculated as 40 per arm. As we expect a maximum of 20% to drop out before the second assessment at 14 weeks after baseline, we aim to recruit a maximum of 100 patients. The trial uses a Bayesian two-arm multistage trial design, where repeated evaluations for efficacy and futility take place after the second assessment CIS-fatigue scores have been observed for 20, 25, 30 and 35 patients in each arm. Frequentist operating characteristics of the trial were evaluated by means of simulation. In the Bayesian model we assumed CIS-fatigue scores to be normally distributed and weakly informative conjugate normal-gamma priors will be used. Parameters for the prior distribution were chosen such that the impact of the prior on the posterior means for the mean and standard deviation for the CIS fatigue scores were negligible. The empirical one-sided significance level was 2.4% and below the desired one-sided significance level of 2.5%. Empirical power was 79% when the standardized effect size was 0.7 (Cohen’s *d*). The expected sample size is 27.4 per arm (54.8 in total) in case of equal means and 31.5 (63 in total) in case standardized effect size is 0.7. A standard two-arm trial without interim analyses would require 43 patients per arm, accounting for 20% drop-out.

### Termination for efficacy or futility

Both at the interim analyses and final analysis, efficacy will be concluded when the posterior probability that the mean CIS-fatigue scores for BCBT are lower than the mean CIS-fatigue scores for usual care exceeds 99%. If this happens at an interim analysis, the trial is stopped and efficacy is concluded. The trial will be stopped for futility as soon as an interim analysis shows that the predictive probability of concluding efficacy at the end of the trial drops below 10%.

### Statistical analysis

The final analysis is conducted on an intention-to-treat basis. Efficacy of the intervention will be concluded based on the posterior probability as outlined in the previous paragraph. Cohen’s *d* will be calculated as an effect size for CIS-fatigue scores where multiple imputation will be used to deal with missing second measurements. Sensitivity analyses will be performed to assess the robustness of the standardized effect size under different imputation strategies.

In addition, analysis of covariance (ANCOVA) will be performed for the secondary outcomes of the second assessment, with the baseline score on the dependent measure as covariate and group allocation as the fixed factor. A *p*-level of 0.05 will be used. Longer-term follow-up effects will also be tested using ANCOVA, with the baseline score on the dependent measure as covariate. Different tumour types will be added to the model as covariates. Cohen’s *d* will be calculated as effect sizes for the secondary outcomes.

## Discussion

Fatigue is the most prevalent symptom of brain tumour patients and is linked to several symptoms, such as depression, sleep disturbances, anxiety, and decreased HRQOL. Patients with diffuse glioma have poor survival and high symptom burden, so the quality of survivorship should be an important factor of treatment. A significant part of care for quality of survivorship is symptom management [63]. Cognitive behavioural interventions seem promising in reducing fatigue severity based on their effectiveness in cancer survivors and palliative cancer patients.

One of the challenges in brain tumour research is the low rate of patient inclusion. Multiple provider reported barriers could explain these low rates, including concerns about the costs and time for patients, frequent hospital visits, suboptimal discussion of possibilities of trial participation and barriers in completing follow-up [64, 65]. This trial tackles some of these barriers by using a blended care intervention with telemedicine limiting visits and travel time. The recruiting clinicians are part of the research team, ensuring a good workflow for patients and clinicians. A researcher will coordinate recruitment, limiting the time spent on recruitment by clinicians. Also, by randomizing patients to a WLC group in the trial, we overcome a potential barrier for patients in enrolling in a trial compared to trials with a care as usual control group. If recruitment is slower than expected after a year, we will include more hospitals for recruitment.

Furthermore, this trial incorporates a Bayesian trial design with a flexible number of patients to be included, ensuring that only the number of patients actually needed to show relevant results will be included. Our trial will include patients with different types of diffuse glioma. This can result in different survival rates, rates of tumour recurrence and dropouts. Subgroup analyses per tumour type may help to explore potentially varying treatment effects.

Potential limitations of the current study design are the inability to blind participants and researchers for the given intervention and the lack of a true placebo condition in the WLC group. These are issues that in general arise in all psychotherapy trials [66]. In this study, patients randomized to the WLC group can be treated for fatigue after the follow-up measurements. It has been hypothesized that a WLC could both result in a placebo and a nocebo response. Provided reasons include that a WLC with future treatment could provide patients with hope for future symptom reduction thus inducing a placebo response, but also that a WLC might result in a nocebo response because the patient has already expressed interest in the therapy and therefore is less motivated to change their behaviour awaiting the promised treatment [66–68]. However, not including a waiting list condition in research in this population is questionable, since patients suffer from severe fatigue with currently no other effective treatment available. Considering this, a WLC seems to be appropriate for this group of vulnerable patients.

The *GRIP on fatigue* trial will provide information on the efficacy of BCBT compared to a WLC in reducing severe fatigue in patients with diffuse glioma. Secondary outcome measures will include different questionnaires concerning HRQOL, subjective cognitive impairments and symptoms such as depression and anxiety. We additionally aim to investigate exploratory measurements that may relate to treatment response and ultimately predict treatment outcome. If proven effective, we aim to offer the intervention as part of usual care for this group of patients.

### Trial status

The protocol reported here is version 8 dated 23 March 2020. Recruitment of patients has started on 11 June 2020 and will continue until at least 1 March 2024. At submission of this paper, the first twelve patients were enrolled in the study.

## Supplementary Information

**Additional file 1: Table S1.** Schedule of enrolment, interventions, and assessments.

## Data Availability

A core anonymised dataset of the primary and secondary outcome measurements will be published online after completion and publication of this trial. The data generated during this trial will also be available from the corresponding author upon reasonable request.

## Abbreviations

BCBT: Blended cognitive behavioural therapy
BDI-PC: Beck Depression Inventory Primary Care
CBT: Cognitive behavioural therapy
CIS: Checklist Individual Strength
CIS-fatigue: Checklist Individual Strength, subscale fatigue severity
CRF: Cancer-related fatigue
GRIP: Guarding quality of survivorship
HRQOL: Health-related quality of life
RCT: Randomized controlled trial
WLC: Waiting list condition
