# Supplementary material for: Reducing severe fatigue in patients with diffuse glioma: a study protocol for an RCT on the effect of blended cognitive behavioural therapy
Source: Trials. 2022 Jul 15;23:568. doi: 10.1186/s13063-022-06485-5 (PMC9287927; doi:10.1186/s13063-022-06485-5)
Supplement: Supplementary file 1 — Additional file 1: Table S1. Schedule of enrolment, interventions, and assessments. [file 13063_2022_6485_MOESM1_ESM.doc]

Table S1. Schedule of enrolment, interventions, and assessments.

|  | **STUDY PERIOD** | | | | | |
| --- | --- | --- | --- | --- | --- | --- |
|  | **Enrolment** | **Allocation** | **Post-allocation** | | | |
| **TIMEPOINT *(in weeks)*** | ***-4*** | ***-2*** | ***0*** | ***1-12*** | ***14*** | ***24*** |
| **ENROLMENT:** |  |  |  |  |  |  |
| **Informed consent** | X |  |  |  |  |  |
| **Eligibility screen** | X |  |  |  |  |  |
| **Allocation** |  | X |  |  |  |  |
| **INTERVENTIONS:** |  |  |  |  |  |  |
| **Blended Cognitive Behavioural Therapy**  Including an assessment of fatigue maintaining cognitive-behavioural factors:   - Sleep wake pattern registration - Actigraphy (1) - *Expectations intervention:* TOEQ (2) - *Fatigue:* Fatigue Catastrophizing Scale (3) - *Focusing on symptoms*: IMQ (4) - *Disease impact*: SIP-sr-sa (5) - *Cognitions*: ICQ (6) - *Posttraumatic Stress*: IES (7) - *Fear progression*: FOP-Q (8) - *Social Support*: SSL-D-N (9) |  |  |  | X |  |  |
| **Waiting list condition** |  |  |  | X |  |  |
| **ASSESSMENTS: all patients** |  |  |  |  |  |  |
| ***Fatigue:* Checklist Individual Strength (10)** | X |  | X |  | X | X |
| ***Depression:* Beck Depression Inventory – PC (11)** | X |  | X |  | X | X |
| ***Depression:* MINI (12)** | X |  |  |  |  |  |
| ***Comorbidities*: Cumulative Illness Rating Scale (13)** |  |  | X |  |  |  |
| ***Fatigue:* Fatigue Severity Scale (14)** |  |  | X |  | X | X |
| ***Self-efficacy:* SES regarding fatigue (15)** |  |  | X |  | X | X |
| ***Sleep quality:* Pittsburgh Sleep Quality Index (16)** |  |  | X |  | X | X |
| ***Health-related QOL:* EORTC QLQ-C30 (17)** |  |  | X |  | X | X |
| ***Brain tumour symptoms:* EORTC QLQ-BN20 (18)** |  |  | X |  | X | X |
| ***Anxiety:* Beck Anxiety Inventory (19)** |  |  | X |  | X | X |
| ***Subjective cognition:* FACT – cog (20)** |  |  | X |  | X | X |
| ***Mastery:* Pearlin Mastery Scale (21)** |  |  | X |  | X | X |
| ***Impaired functioning:* WSAS(22)** |  |  | X |  | X | X |
| ***Happiness:* SHQ (23)** |  |  | X |  | X | X |
| ***Functional impairment:* KPS (24)** |  |  | X |  | X | X |
| **Neuropsychological assessment** |  |  | X |  | X |  |
| **ASSESSMENTS: only in the BCGT group** |  |  |  |  |  |  |
| ***Satisfaction:* Patient and therapist satisfaction** |  |  |  |  | X |  |
| ***System:* System Usability Scale (25)** |  |  |  |  | X |  |
| **COLLECTED DATA: only in the BCGT group** |  |  |  |  |  |  |
| ***System:* Usage data of the web-based program** |  |  |  |  | X |  |
| ***Time therapists*: Time invested per patient** |  |  |  |  | X |  |
| **EXPLORATORY ASSESMENTS** |  |  |  |  |  |  |
| **Resting-state functional MRI and diffusion MRI** |  |  | X |  | X |  |
| **Magnetoencephalography** |  |  | X |  | X |  |
| **Neurological Assessment in Neuro-Oncology (26)** |  |  | X |  | X |  |
| **BCBT**, blended cognitive behavioural Therapy; **BN20**, brain tumour module; **EORTC**, European Organization for Research and Treatment for Cancer; **FACT-cog**, Functional Assessment of Cancer Therapy - cognitive function issues; **FOP-Q**, Fear of Progression Questionnaire; **ICQ**, Illness Cognition Questionnaire; **IES**, Impact Event Scale**;** **IMQ**, Illness Management Questionnaire factor III; **KPS**, Karnofsky performance status scale; **MINI**, Mini-International Neuropsychiatric Interview-depressive disorder **PC**, primary care; **QLQ-C30**, Quality of Life of Cancer Patients; **QOL**, quality of life; **SES**, Self-efficacy Scale; **SHQ**, Subjective Happiness Questionnaire; **SIP-sr-sa**, Sickness Impact Profile subscale sleep and rest and social activities; **SSL-D-N**, social support list subscale discrepancies and negative interactions; **TOEQ**, Treatment Outcome Expectation Questionnaire; **WSAS**, Work and Social Adjustment Scale; | | | | | | |

## REFERENCES

1. Gresham G, Schrack J, Gresham LM, Shinde AM, Hendifar AE, Tuli R, et al. Wearable activity monitors in oncology trials: Current use of an emerging technology. Contemporary Clinical Trials. 2018;64:13-21.

2. Heins MJ, Knoop H, Bleijenberg G. The role of the therapeutic relationship in cognitive behaviour therapy for chronic fatigue syndrome. Behav Res Ther. 2013;51(7):368-76.

3. Jacobsen PB, Andrykowski MA, Thors CL. Relationship of catastrophizing to fatigue among women receiving treatment for breast cancer. J Consult Clin Psychol. 2004;72(2):355-61.

4. Ray C, Weir W, Stewart D, Miller P, Hyde G. Ways of coping with chronic fatigue syndrome: development of an illness management questionnaire. Soc Sci Med. 1993;37(3):385-91.

5. Jacobs HM, Luttik A, Touw-Otten FW, de Melker RA. The sickness impact profile; results of an evaluation study of the Dutch version. Ned Tijdschr Geneeskd. 1990;134(40):1950-4.

6. Evers AW, Kraaimaat FW, van Lankveld W, Jongen PJ, Jacobs JW, Bijlsma JW. Beyond unfavorable thinking: the illness cognition questionnaire for chronic diseases. J Consult Clin Psychol. 2001;69(6):1026-36.

7. van der Ploeg E, Mooren TT, Kleber RJ, van der Velden PG, Brom D. Construct validation of the Dutch version of the impact of event scale. Psychol Assess. 2004;16(1):16-26.

8. Goebel S, Mehdorn HM. Fear of disease progression in adult ambulatory patients with brain cancer: prevalence and clinical correlates. Support Care Cancer. 2019;27(9):3521-9.

9. van Sonderen E. Sociale Steun Lijst-Interacties (SSL-I) en Sociale Steun Lijst - Discrepanties (SSL-D). Groningen: Noordelijk Centrum voor Gezondheidsvraagstukken; 1993.

10. Worm-Smeitink M, Gielissen M, Bloot L, van Laarhoven HWM, van Engelen BGM, van Riel P, et al. The assessment of fatigue: Psychometric qualities and norms for the Checklist individual strength. J Psychosom Res. 2017;98:40-6.

11. Beck AT, Guth D, Steer RA, Ball R. Screening for major depression disorders in medical inpatients with the Beck Depression Inventory for Primary Care. Behav Res Ther. 1997;35(8):785-91.

12. Sheehan DV, Lecrubier Y, Sheehan KH, Amorim P, Janavs J, Weiller E, et al. The Mini-International Neuropsychiatric Interview (M.I.N.I.): the development and validation of a structured diagnostic psychiatric interview for DSM-IV and ICD-10. J Clin Psychiatry. 1998;59 Suppl 20:22-33;quiz 4-57.

13. Parmelee PA, Thuras PD, Katz IR, Lawton MP. Validation of the Cumulative Illness Rating Scale in a geriatric residential population. J Am Geriatr Soc. 1995;43(2):130-7.

14. Krupp LB, LaRocca NG, Muir-Nash J, Steinberg AD. The fatigue severity scale. Application to patients with multiple sclerosis and systemic lupus erythematosus. Archives of neurology. 1989;46(10):1121-3.

15. Prins JB, Bleijenberg G, Bazelmans E, Elving LD, de Boo TM, Severens JL, et al. Cognitive behaviour therapy for chronic fatigue syndrome: a multicentre randomised controlled trial. The Lancet. 2001;357(9259):841-7.

16. Mollayeva T, Thurairajah P, Burton K, Mollayeva S, Shapiro CM, Colantonio A. The Pittsburgh sleep quality index as a screening tool for sleep dysfunction in clinical and non-clinical samples: A systematic review and meta-analysis. Sleep Med Rev. 2016;25:52-73.

17. Aaronson NK, Ahmedzai S, Bergman B, Bullinger M, Cull A, Duez NJ, et al. The European Organization for Research and Treatment of Cancer QLQ-C30: a quality-of-life instrument for use in international clinical trials in oncology. J Natl Cancer Inst. 1993;85(5):365-76.

18. Osoba D, Aaronson NK, Muller M, Sneeuw K, Hsu MA, Yung WK, et al. The development and psychometric validation of a brain cancer quality-of-life questionnaire for use in combination with general cancer-specific questionnaires. Qual Life Res. 1996;5(1):139-50.

19. Ke Y, Ng T, Yeo HL, Shwe M, Gan YX, Chan A. Psychometric properties and measurement equivalence of the English and Chinese versions of the Beck Anxiety Inventory in patients with breast cancer. Support Care Cancer. 2017;25(2):633-43.

20. Cheung YT, Lim SR, Shwe M, Tan YP, Chan A. Psychometric properties and measurement equivalence of the English and Chinese versions of the functional assessment of cancer therapy-cognitive in Asian patients with breast cancer. Value in health : the journal of the International Society for Pharmacoeconomics and Outcomes Research. 2013;16(6):1001-13.

21. Krokavcova M, Nagyova I, van Dijk JP, Rosenberger J, Gavelova M, Middel B, et al. Mastery, functional disability and perceived health status in patients with multiple sclerosis. European journal of neurology. 2008;15(11):1237-44.

22. Thandi G, Fear NT, Chalder T. A comparison of the Work and Social Adjustment Scale (WSAS) across different patient populations using Rasch analysis and exploratory factor analysis. J Psychosom Res. 2017;92:45-8.

23. Lyubomirsky S, Lepper HS. A Measure of Subjective Happiness: Preliminary Reliability and Construct Validation. Social Indicators Research. 1999;46(2):137-55.

24. Schaafsma J, Osoba D. The Karnofsky Performance Status Scale re-examined: a cross-validation with the EORTC-C30. Qual Life Res. 1994;3(6):413-24.

25. Bangor A, Kortum PT, Miller JT. An Empirical Evaluation of the System Usability Scale. International Journal of Human–Computer Interaction. 2008;24(6):574-94.

26. Nayak L, DeAngelis LM, Brandes AA, Peereboom DM, Galanis E, Lin NU, et al. The Neurologic Assessment in Neuro-Oncology (NANO) scale: a tool to assess neurologic function for integration into the Response Assessment in Neuro-Oncology (RANO) criteria. Neuro Oncol. 2017;19(5):625-35.
